# Crystallized TiO_2_ Nanosurfaces in Biomedical Applications

**DOI:** 10.3390/nano10061121

**Published:** 2020-06-06

**Authors:** Metka Benčina, Aleš Iglič, Miran Mozetič, Ita Junkar

**Affiliations:** 1 Department of Surface Engineering and Optoelectronics, Jožef Stefan Institute, Jamova 39, SI-1000 Ljubljana, Slovenia; miran.mozetic@ijs.si (M.M.); ita.junkar@ijs.si (I.J.); 2 Laboratory of Physics, Faculty of Electrical Engineering, University of Ljubljana, Tržaška 25, SI-1000 Ljubljana, Slovenia; ales.iglic@fe.uni-lj.si; 3 Faculty of Medicine, University of Ljubljana, Zaloška 9, SI-1000 Ljubljana, Slovenia

**Keywords:** titanium oxide, nanostructure, crystalline phase, biocompatibility, surface modification

## Abstract

Crystallization alters the characteristics of TiO_2_ nanosurfaces, which consequently influences their bio-performance. In various biomedical applications, the anatase or rutile crystal phase is preferred over amorphous TiO_2_. The most common crystallization technique is annealing in a conventional furnace. Methods such as hydrothermal or room temperature crystallization, as well as plasma electrolytic oxidation (PEO) and other plasma-induced crystallization techniques, present more feasible and rapid alternatives for crystal phase initiation or transition between anatase and rutile phases. With oxygen plasma treatment, it is possible to achieve an anatase or rutile crystal phase in a few seconds, depending on the plasma conditions. This review article aims to address different crystallization techniques on nanostructured TiO_2_ surfaces and the influence of crystal phase on biological response. The emphasis is given to electrochemically anodized nanotube arrays and their interaction with the biological environment. A short overview of the most commonly employed medical devices made of titanium and its alloys is presented and discussed.

## 1. Introduction

Titanium-based alloys are considered as more biocompatible metallic biomaterials compared to stainless steel, cobalt–nickel, and chromium alloys, due to their low ion release when exposed to human body liquids [1], low elastic modulus [2], and relatively high strength to density ratio, as well as corrosion resistance [3]. The biocompatibility of titanium (Ti) arises from its high reactivity with oxygen when Ti is exposed to air, which leads to the formation of a chemically stable passivating oxide layer. The naturally formed oxide layer (of 2–5 nm thickness) prevents surface corrosion and makes the material bioinert. As the biological response to biomaterial is mainly governed by the biomaterial surface properties, it is of primary importance to condition the surface appropriately in order to obtain the desired surface interaction with the surrounding cells and proteins. Immediately after the exposure of the biomaterial surface to the biologic environment, the so-called “race for the surface” begins and the proteins are the ones that reach the surface first. The type and amount of proteins, as well as their conformational state, further influence and guide cell adhesion. Thus, surface properties, in terms of protein adhesion, should also be taken into consideration. Moreover, with the development of nanotechnology, it has been shown that surface features significantly influence the amount and type of adhered proteins, as well as their conformational state, which further dictate their interaction with cells. Besides other characteristics, such as surface morphology, chemistry, charge, wettability, and the crystal structure of TiO_2_ nanosurfaces highly affect the material’s bio-performance. For example, nanostructured titanium surfaces in the form of TiO_2_ nanotubes increase bone growth/regeneration, are antibacterial, and reduce inflammation [4,5,6,7,8,9]. Besides, it has been shown that the annealing temperature and crystal phase of TiO_2_ nanotubes is highly related to platelet adhesion and activation [10]. Huang et al. [11] demonstrated that TiO_2_ nanotubes with an anatase crystal structure are more susceptible to platelet adhesion, while rutile phase TiO_2_ nanotubes reduced platelet adhesion and activation. A recent study by Li et al. [12] on the three types of titania nanostructures (nanowires, nanonests, and nanoflakes) obtained by hydrothermal treatment showed enhanced osteointegration and desired macrophage responses, mainly due to nanotopography, as well as surface-induced crystallization. It was also demonstrated that hydrothermally treated titanium surfaces significantly reduce bacterial adhesion, mainly due to the formation of an appropriate surface topography [13,14].

Plasma technologies also hold great potential in the medical field, as the surface properties of biomaterials (e.g., sterilization, decontamination, surface finishing) can be easily modified without influencing the bulk attributes of the material [15,16,17,18,19]. Plasma is able to modify surface properties in terms of surface wettability, chemistry, surface charge, morphology, and crystallinity due to the interaction of the plasma species with biomaterial surfaces. Our previous study [20] indicates that the combination of nanotopography (TiO_2_ nanotubes) and plasma surface modification influenced the growth of endothelial cells (ECs), smooth muscle cells (SMCs), and platelet adhesion. The selective growth of ECs over SMCs was observed, while platelet adhesion was significantly reduced. This was achieved where both electrochemical anodization (nanostructured surface) and gaseous plasma treatment (the formation of high quality titanium oxide) were combined.

In the present review, the influence of different crystalline forms on TiO_2_ nanosurfaces, with an emphasis on TiO_2_ nanotubes, on biological performance will be presented. In addition, various crystallization methods and the mechanisms involved will be discussed.

## 2. Crystal Structures of TiO_2_

**Figure 1:**
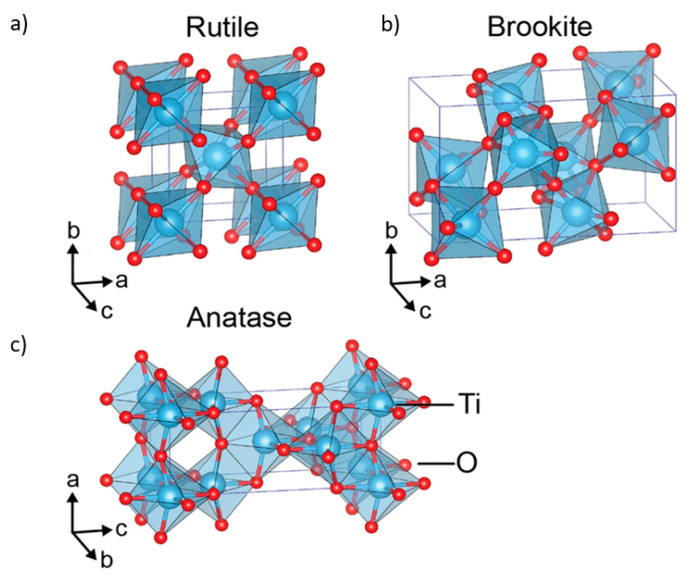
Crystal structures of TiO_2_: (**a**) rutile; (**b**) anatase; (**c**) brookite; Reproduced with permission from from [27]. Scientific Reports, 2017.

TiO_2_ material appears in various polymorphs that are built of the same fundamental TiO_6_ polyhedral building units but are connected differently [21]. The crystal structures of titania differ by the spatial arrangement of the TiO_6_ octahedral building blocks. In all polymorphs of titanium, a titanium cation is six-fold coordinated to the surrounding oxygen anions, forming distorted TiO_6_ octahedra joined by sharing the octahedral edges. Anatase, rutile, and brookite crystal structures are presented in Figure 1, while other TiO_2_ crystal phases have also been reported (TiO_2_ II or srilankite, cubic fluorite-type, pyrite-type, monoclinic baddeleyite-type, and cotunnite-type polymorphs), however, their applications are limited [22]. Rutile has a tetragonal structure and contains six atoms per unit cell and it is the most stable polymorph of TiO_2_. Anatase has a tetragonal structure, in which the octahedra are linked together through corner sharing [23]. Brookite is the orthorhombic version of TiO_2_, in which the octahedra share both edges and corners [24]. Anatase and brookite are meta-stable at the bulk form and readily transform to rutile when heated [25]. However, at the nanoscale, anatase and brookite are stable due to their smaller surface energy [26] and transform into the rutile phase only after reaching a certain nanoparticle size (more than 14 nm) [22].

## 3. Crystallization Process

The crystallization of amorphous solid nanosurfaces can be explained as a process in which the atoms are organized into a crystal through nucleation (the appearance of a crystalline phase) and crystal growth (the appearance of a crystalline phase from either a supercooled liquid or a supersaturated solvent). The mechanism of the crystallization of amorphous TiO_2_ nanosurfaces, as well as the kinetics and mechanisms of the solid state transformation from anatase to rutile, can be found elsewhere [28,29,30]. Briefly, the crystallization of TiO_2_ nanosurfaces is governed by an increased temperature; the amorphous material is transformed to a lower temperature phase—anatase [28]—and upon further heating to 400–1200 °C [31,32], into a rutile phase. Different crystallization conditions affect the crystallization mechanism. For instance, the physical characteristics of the transformation in an air atmosphere differ from those in liquid, in which dissolution steps are involved [28]. In the following sections, various crystallization methods and mechanisms of TiO_2_ nanosurfaces are presented, with an emphasis on plasma-induced crystallization performed in our previous study [33].

### 3.1. Crystallization by Annealing in a Gaseous Atmosphere

Heat treatment in a conventional furnace is a commonly applied method of the crystallization of TiO_2_ nanosurfaces. However, heat treatment in a conventional furnace is time-consuming; in order to achieve the transformation from amorphous to anatase, a mixture of anatase/rutile, and rutile crystal structures, TiO_2_ nanotubes synthesized by an electrochemical anodization method should undergo annealing for at least 2 h at 450 °C, 2 h at 550 °C [34], and 2 h at 800 °C [35], respectively. Bakri et al. [36] showed that TiO_2_ films obtained by the sol–gel dip-coating method crystallize in an anatase crystal structure at the annealing temperature of 300 °C and transform to a rutile phase at 900 °C. Catauro et al. [37], on the other hand, demonstrated that higher annealing temperatures induced the formation of an anatase and rutile mixture with different contents of each. The relative amount of anatase formation in the final material is higher for the samples anodized with a higher voltage compared to the TiO_2_ nanotubes anodized at a lower voltage [38]. Sangani et al. [39] demonstrated that the addition of metals, such as Au, to the sol–gel derived TiO_2_ thin films also decreases the crystallization temperatures. Elevated temperatures are, however, responsible for the atoms’ rearrangements in a TiO_2_ lattice. The initial crystal phase of TiO_2_, upon annealing, is generally anatase [40,41]. The reason for this could be a more feasible arrangement of the short-range ordered TiO_6_ octahedra into the long-range ordered anatase structure due to the less constrained construction of anatase in relation to rutile [42]. However, from a thermodynamic point of view, the accelerated recrystallization of anatase could be due to the lower surface free energy of this crystal phase [43,44].

### 3.2. Crystallization by the Hydrothermal Process

Another intriguing low-temperature crystallization procedure in aqueous media is a hydrothermal treatment in a Teflon-lined stainless autoclave containing a small amount of water, which turns into water vapor at elevated temperatures, which is responsible for the crystallization. Crystallite growth is induced by the dissolution–precipitation mechanism, in which randomly distributed TiO_6_ octahedra are rearranged with the assistance of water; fine crystals are formed when the nucleation rate increases, but the epitaxial crystal growth mechanism is inhibited by the water, since the rapid nucleation leaves little material for further growth [45]. The crystallization can also be catalyzed by pressure and mineralizing agents [46]. Liu et al. [47] employed this solid–gas method for the transition of amorphous TiO_2_ nanotubes to an anatase crystal phase at a temperature of less than 180 °C for 4 h with only 0.3 mL water added to the autoclave.

### 3.3. Room Temperature Crystallization

**Figure 2:**
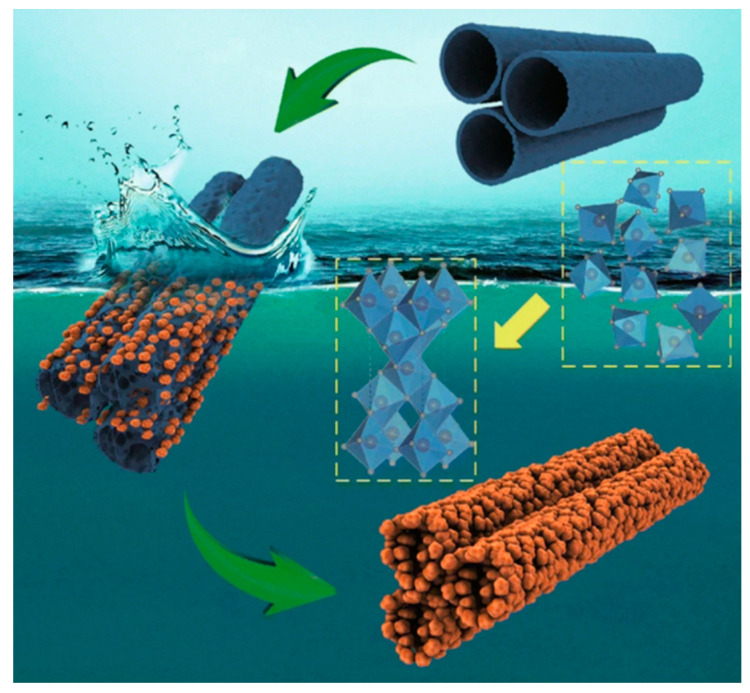
Schematic illustration of the crystallization process in water. Reproduced with permission from [50]. Nano-Micro Letters, 2018.

Lamberti et al. [48] demonstrated a near-room temperature (50 °C) crystallization process of TiO_2_ nanotubular layers. Amorphous TiO_2_ was rearranged to an anatase phase after 30 min of exposure to water vapor. The authors suggested that the condensed water acted as a catalyst and favored the rearrangement of the TiO_6_ octahedra. However, such a crystallization procedure induces the formation of crystals at the outer and inner walls of the nanotubes, which leads to structural transformation of the nanotubes to nanorods. John K. et al. [49] reported on the room temperature crystallization of TiO_2_ nanotubes by the application of alternating voltage square pulses. Crystallization occurs without any direct dissolution of TiO_6_ octahedra due to the rapid application of a negative pulse followed by a positive pulse. When the positive pulse is applied to the TiO_2_ nanotube electrode in water, the OH- ions accumulate on its surface. Then, the lone electron of OH- ions form bridge bonds with the hydroxyl ions present in the two adjacent TiO_6_ octahedra of the TiO_2_ nanotubes. The negative pulse, on the other hand, initiates the attraction of hydrogen ions to the surface of the TiO_2_ nanotube electrode. The hydrogen ions trigger the dehydration of the TiO_6_^2−^ octahedra of the TiO_2_ nanotubes and results in the formation of octahedra shared by edges. This process continues until the anatase crystal phase is formed [49,50]. Krengvirat et al. [51] demonstrated the crystallization of TiO_2_ naotubular arrays by the immersion of amorphous TiO_2_ nanotube arrays into hot water (70–90 °C) under ambient pressure and neutral pH. It seems that the alteration of morphology during such water treatment is inevitable; the transformation of uniform nanotubes walls to granular anatase TiO_2_ nanoparticles occurs, as schematically presented in Figure 2.

### 3.4. Plasma-Induced Crystallization

Plasma technologies have gained significant importance in the medical field for improving the surface properties of biomaterials. Plasma is actually an excited ionized gas that is composed of natural species, charged particles (ions, electrons), and electric fields, as well as ultraviolet radiation, which with a high efficiency alter the surface properties of biomaterials due to the interaction of plasma species with the surfaces. The main advantage of plasma modification is that only the top surface layer is modified, which enables the preservation of the bulk attributes of the material. By plasma surface modification surface chemistry, morphology (on the nanoscale), wettability and surface charge, as well as crystallinity, can be altered in order to obtain the desired biological response. Thus, plasma surface modification is one of the methods to be used in order to improve surface properties for the desired biological response. The plasma-based technologies may, by their mechanisms of modification, significantly differ but all can, to some extent, offer the formation of anatase or anatase/rutile structures on the surface of titanium. The most common commercially employed technique for the surface modification of implants is plasma spraying. Plasma spraying (PS) uses high temperatures in order to melt powder particles, which are plasma sprayed on the substrate, which enable the transformation of TiO_2_ metastable phases [52]. In this case, different types of plasma spraying may be used (atmospheric plasma spraying, vacuum plasma spraying, water-stabilized plasma spraying, etc.). Another interesting and commercially available approach used in the surface treatment of biomaterials is plasma electrolytic oxidation (PEO), also known as micro-arc plasma (MPA). It is a combination of the high voltage spark and electrochemical oxidation and it usually enables the good adhesion and homogeneity of oxide coatings. However, in this case, the electrolyte has to be used and sometimes this can present environmental issues (depending on the type of electrolyte used) but it can, at the same time, provide numerous treatment possibilities, as many different types and combinations of electrolytes may be employed, and some of them even enable the enrichment of the surface with biocompatible elements (i.e., calcium and phosphorus). The phase composition of PEO titanium oxide coatings (amorphous, crystalline) strongly depends on the processing conditions (voltage, current density, electrolyte, time etc.), while high temperatures, which locally occur at the surface, are correlated with the growth of nanocrystals [53,54,55,56,57,58,59,60,61,62]. Titanium coatings may also be deposited by chemical vapor deposition (CVD) or physical vapor deposition (PVD), which can also be plasma enhanced (such as plasma enhanced chemical vapor deposition—PECVD), and treatment with high energy ions (ion implementation). The ion beam implementation processes may provide advantages, such as the concentration and depth distribution of impurities, which can be controlled with high accuracy, and there are no sharp interfaces between the implanted layer and substrate, which is typical in many other coating procedures. This also prevents the risk of coating delamination. Recently, a novel approach to use the “low temperature” plasma treatment to induce the phase transformation of amorphous TiO_2_ oxide layers to a crystalline phase was proposed by an atmospheric pressure radio-frequency (RF) plasma (30 min treatment at 60 W) [63], where the measured temperature of the substrate was assumed not to be above 322 °C. The RF low pressure nitrogen plasma was recently also shown to initiate phase transformation at low temperatures and thus lower operational costs [64]. The so-called low temperature plasma-induced crystallization methods on Ti-based nanosurfaces are reviewed in Table 1.

**Figure 3:**
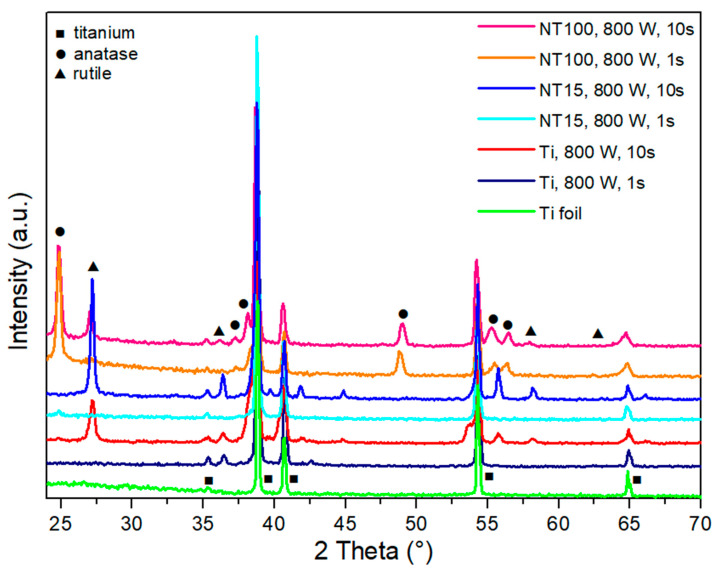
X-ray diffraction (XRD) patterns of untreated Ti foil, Ti foil exposed to plasma of 800 W for 1 s and 10 s, TiO_2_ nanotubes with a 15 nm diameter (NT15) exposed to plasma of 800 W for 1 s and 10 s, and TiO_2_ nanotubes with a 100 nm diameter (NT100) exposed to plasma of 800 W for 1 s and 10 s. Ti = titanium, A = anatase crystal structure, R = rutile crystal structure.

The mechanism of low temperature plasma-induced crystallization is rather unknown. Ohsaki et al. [65] crystallized the sol–gel-derived TiO_2_ thin films within a few minutes by non-thermal plasma processing. The suggested mechanisms of such crystallization could be the excitations by the radio frequency (RF) electromagnetic field and not the plasma itself. Meanhhile, Krylov et al. [67] interestingly observed that amorphous TiO_2_ prepared by sol–gel hydrolysis experienced photoinduced crystallization under UV irradiation in a few hours. The one-nanometer-thick rutile shell was formed around an amorphous core, which gives evidence that in plasma a similar phenomenon can take place, as plasma is also a source of UV radiation. Meanwhile, in another study by An et al. [66], it is suspected that the formation of the crystalline grains of BaTiO_3_ occurs due to the energy provided by plasma ion bombardment. Kramer et al. [68] suggested that the silicon nanoparticles exceed the gas temperature in non-thermal plasmas to the point of a temperature sufficient for crystallization (up to 700 K/427 °C). Measurements showed that the average temperature of the silicon nanoparticles exposed to plasma was close to the gas temperature. According to the authors, the crystallization of the nanoparticles by non-thermal plasma occurs due to the electron ion recombination and the reactions of radicals on the nanoparticle’s surface [68]. Similarly, Lopez et al. [69] demonstrated that plasma exposure allowed for the crystallization of silicon nanoparticles due to the heating of the nanoparticles to a temperature of 1100 K/827 °C. The most likely explanation is that the synergistic effects in plasma take place, which not only depend on plasma treatment conditions, but also on the morphology of the modified surface. This was also confirmed recently by our study [33], in which the transition from amorphous to anatase and rutile phase was reached depending on surface morphology (diameter and length) of TiO_2_ nanotubes. Treatment with oxygen plasma of pure titanium foils (naturally formed TiO_2_ layers on the top surface), and TiO_2_ nanotubes with different lengths and diameters formed on the surface of a Ti substrate with the process of electrochemical anodization, initiated the transition of the amorphous phase to an anatase/rutile crystal structure depending on the plasma treatment conditions. Interestingly, it was shown that TiO_2_ nanotubes with a 100 nm diameter and a length of about 2.5 µm more easily crystallize into the mixture of rutile and anatase crystal phases at a high output power of plasma compared to TiO_2_ nanotubes with a 15 nm diameter and a length of about 0.2 µm. As shown in Table 2, treatment at 800 W for 10 s enabled the formation of a rutile structure on TiO_2_ nanotubes with a 15 nm diameter, while the mixture of anatase and rutile phases was detected in the case of TiO_2_ nanotubes with a 100 nm diameter. However, in the case of plain Ti foil (a TiO_2_ surface without artificially formed nanotopography) the rutile (2θ = 36.5°) and anatase peaks (2θ = 37.5°) were detected after 1 s and 10 s treatments at 800 W, as can be seen in Figure 3.

**Figure 4:**
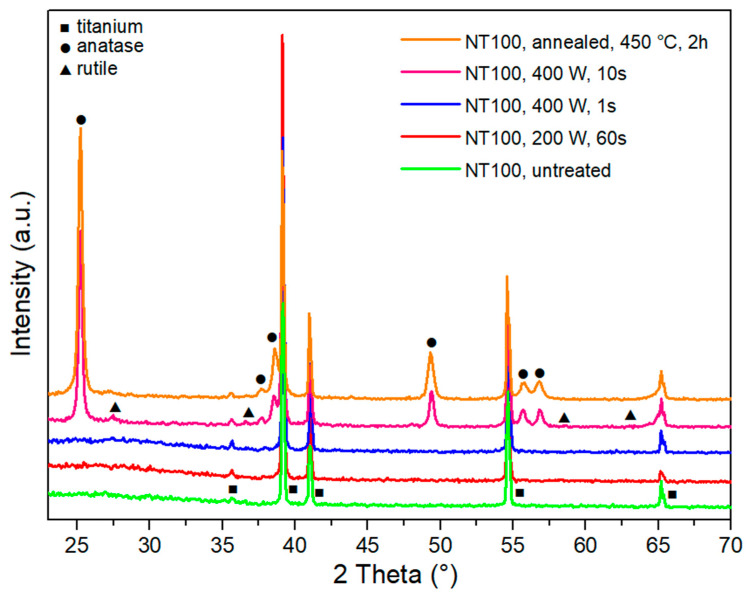
X-ray diffraction (XRD) patterns of untreated TiO_2_ nanotubes, TiO_2_ nanotubes exposed to plasma of 200 W for 60 s, 400 W for 1 s and 10 s, and TiO_2_ nanotubes after annealing in a furnace at 450 °C for 2 h. NT100 = TiO_2_ nanotubes with a 100 nm diameter, Ti = characteristic peak for titanium, A = anatase crystal structure, R = rutile crystal structure.

Furthermore, the influence of different plasma output powers was studied and the results are presented in Figure 4. The formation of an anatase phase is observed in the case of plasma treatment at higher powers (400 W to 800 W), while a rutile structure was detected only in the case of plasma treatment at higher powers (800 W for 10 s). UV plasma radiation was present in all cases, however, it seems that the predominant role in plasma-induced crystallization is due to temperature effects attributed to the interaction of radicals and ions with the surface (sample heating). However, other synergistic effects of plasma should be considered, as according to our study, the gas temperature of RF oxygen plasma measured at all output powers (200–800 W) was well below the thermal crystallization temperature, and the temperature of the sample could, in the most harsh treatment conditions, (800 W for 10 s) rise above 1300 °C [33].

Moreover, employing a plasma treatment to hydrothermally prepared nanostructured titanium surfaces enabled the formation of denser and higher quality oxygen layers and the removal of contaminants, like carbon, from the surface, which was shown to influence the biological response (bacterial adhesion, osteoblast cell proliferation) [70,71,72].

## 4. Influence of TiO_2_ Crystal Structures on Material Characteristics

The annealing/crystallization of TiO_2_ nanotubes affects the surface properties, such as wettability, surface chemistry, and even morphology [48,73]. For instance, TiO_2_ nanotubes annealed in the furnace at high temperatures are exposed to changes in morphology [35,74], which may influence their biocompatibility.

### 4.1. Influence on Wettability

The wettability of biomaterials is one of the most important surface features which influences the biological response, thus the evaluation of the water contact angle (WCA) of biomaterials is one of the most quick and simple methods for the evaluation of surfaces properties. It has been shown that annealed TiO_2_ nanotubes with an anatase crystal structure have a more hydrophilic character compared to amorphous samples formed with electrochemical anodization [48,75]. Moreover, the annealing of TiO_2_ nanotubes increases their corrosion resistance in bioliquids and improves their electrochemical stability [76,77]. Additionally, the so-called “ageing” of surfaces after storage is another important feature, which should be considered in the final application of biomaterials, as even small changes in wettability may alter the biological response. It was shown by our study [78] that TiO_2_ nanotubes have different wettabilities after storage in air, depending on their treatment procedure. As-formed amorphous nanotubes exhibit a hydrophilic character even ten weeks after fabrication (Table 3). The water contact angle (WCA) measured after 10 weeks of ageing shows the most prominent changes in wettability. In this case, the highest wettability was observed in the case of plasma-treated anatase/rutile nanotubes (NTs), followed by annealed anatase NTs and plasma-treated amorphous NTs. The freshly prepared NTs were practically hydrophobic after 10 weeks of ageing, as the contact angle increased from an initial 4° to about 73°, but still less than plain Ti foil (98°). The more hydrophilic character of plasma-treated TiO_2_ nanotubes could be due to a different crystal phase, however, another explanation is that the hydroxyl groups, as well as the formation of oxygen vacancies on the TiO_2_ surface due to plasma functionalization, are responsible for the improved wettability. In our study [79], we also showed that the TiO_2_ nanotubes’ diameter (15, 50 and 100 nm), due to their specific size, affects the interaction of water with the titanium dioxide surface.

### 4.2. Influence on Mechanical/Tribological Properties

Titanium implants are subjected to micromovements under loading conditions in an aggressive biological environment, which causes tribocorrosion. It has been shown that the annealing of TiO_2_, and subsequently the formation of anatase and rutile crystalline phases, influences both mechanical and tribological properties [80,81]. Alves et al. [82] showed that wear resistance is dependent on the oxide layer hardness and adhesion strength to the Ti substrate, which is significantly influenced by the formation of a nano-thick oxide film formed at the interface region due to crystallization. Almeida Fontes et al. [83] showed that the annealing temperature and crystalline phases of the TiO_2_ nanotubes highly affects the mechanical and tribological properties of the oxide layer. TiO_2_ nanotubes with an anatase crystal structure have mechanical properties (Hardness (H) and an elastic modulus (E)) lower than the Ti substrate, which present abrasive and adhesive wear and reveal the substrate during sliding in the tribocorrosion test. TiO_2_ nanotubes with the presence of a rutile layer presented higher values of elastic modulus, higher hardness, and consequently a higher tribocorrosion resistance.

### 4.3. Influence on Surface Chemistry

The top surface layer chemical composition is also one of the important features influencing the biological response to biomaterials. X-ray diffraction (XRD) provides information about the crystalline phase in TiO_2_ surfaces, however, for the analysis of the top surface layer and chemical information of the surface, higher surface sensitivity is needed. For example, a more powerful tool for surface analysis is X-ray photoemission spectroscopy (XPS), where information about the surface chemistry comes from the top surface layer (about 4–6 nm) or Time-of-flight secondary ion mass spectrometry (ToF-SIMS) which enables the identification of the molecular structure of atomic surfaces (1 nm) with high spatial resolution (50–100 nm). Both techniques are especially valuable for the detection of the chemical composition of the surface, which is highly relevant in the case of biomaterials, as the top surface layer interacts with the biological environment. In the case of metal surfaces, organic contamination due to surface finishing procedures is very common and could affect the biological response [72]. Moreover, in the case of TiO_2_ nanotubes, a remnant of fluorine (F) is usually present on the surface due to the electrolytes containing hydrofluoric acid used for anodization. Fluorine exhibits biological toxicity and is therefore not desirable in bio-applicative materials. According to XPS results, F is present on an electro-anodized nanotubular surface from about 3.0 at.% to 6.4 at.%, depending on the nanotube’s diameter. It seems that the F content increases with the nanotube’s diameter, as seen in Table 4. After plasma treatment at 200 W for 60 s, the surface is still in an amorphous phase, but plasma seems to remove F from the top surface, as the F content decreases from 6 at.% to about 1.5 at.%. When the surface is heated in a furnace, no F was detected on the surface, and in this case, the nanotubes were already in the anatase form. Thus, altering the crystal structure, as well as the top chemistry by heat treatment, could beneficially influence the biological response.

### 4.4. Influence on Surface Topography

**Figure 5:**
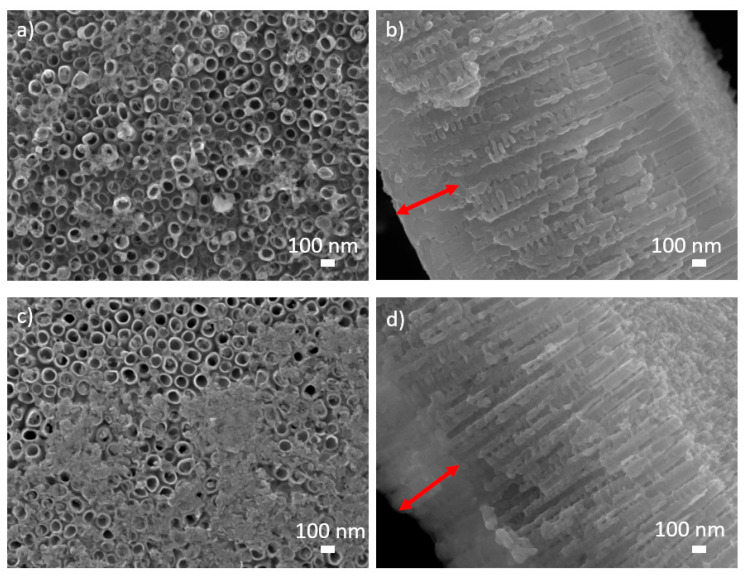
SEM images (above: top view and below: cross-sectional view) of TiO_2_ nanotubes of 100 nm in diameter treated with oxygen plasma at (**a**,**b**) 800 W for 1 s and (**c**,**d**) 800 W for 10 s. The red arrow indicates the destroyed bottom layer of nanotubes.

The alteration of crystal structure influences the topography of nanofeatures [74]. In our recent study [33], the TiO_2_ nanotubes formed by electrochemical anodization on the surface of titanium sheets were crystallized to anatase and a mixture of anatase and rutile crystal phases after exposure to highly reactive oxygen plasma (Figure 3 and Figure 4). The analysis of the crystallized TiO_2_ nanotubes with SEM revealed the transformation of the nanotubular structure to a compact layer at the nanotube/Ti foil interface. It was previously reported that the formation of a rutile oxide layer is initiated at the metal–nanotube interface during the annealing stage [84], and therefore the changes in crystal structure firstly occur at the bottom layer of the nanotubes. In Figure 5, the plasma-treated samples at 800 W for 10 s (anatase/rutile mixture) and 800 W for 1 s (anatase) are presented. It can be observed that the bottom layer of the nanotubes is destroyed and the formation of an oxide layer can be detected (red arrow).

That phenomenon has also been observed by Das et al. [84] and it is presumably due to the exposure of nanotubes to high temperatures. In other reports [85,86], the same loss of nanotubular structure after annealing at high temperatures was also observed.

**Figure 6:**
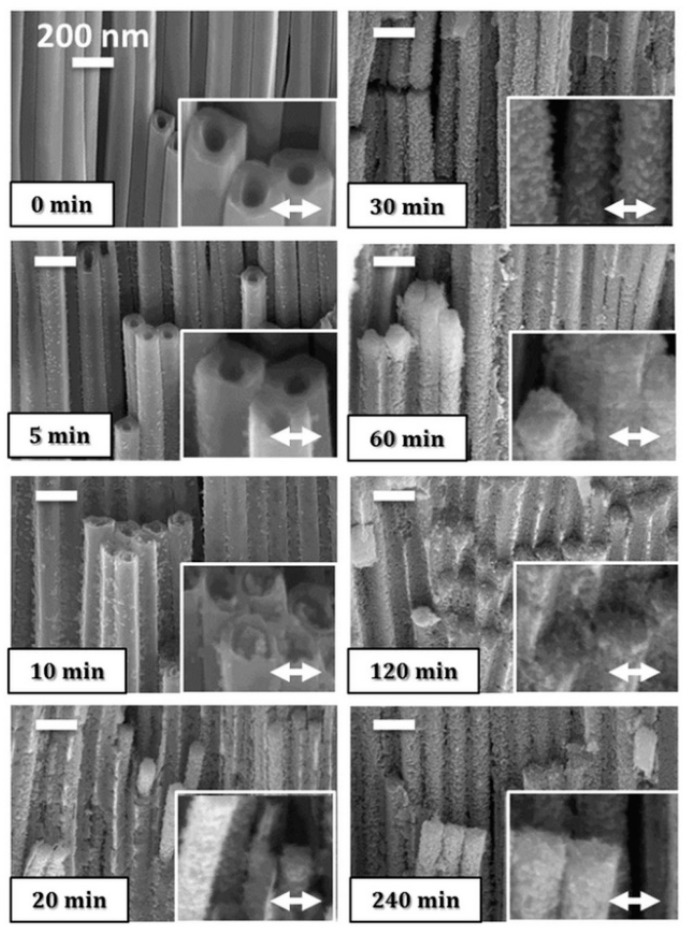
SEM images presenting the alterations of the morphology of a TiO_2_ nanotubular array as a function of the water vapor exposure time (0–240 min). Reproduced with permission from [48]. Scientific Reports, 2014.

Similarly, Lamberti el al. [48] reported on the structural transformation of amorphous nanotubes to nanorods with an anatase phase by the process of low thermal crystallization after exposure to water vapor. Such a crystallization procedure causes the formation of crystals at the outer and inner walls of the nanotubes, as presented in Figure 6.

**Figure 7:**
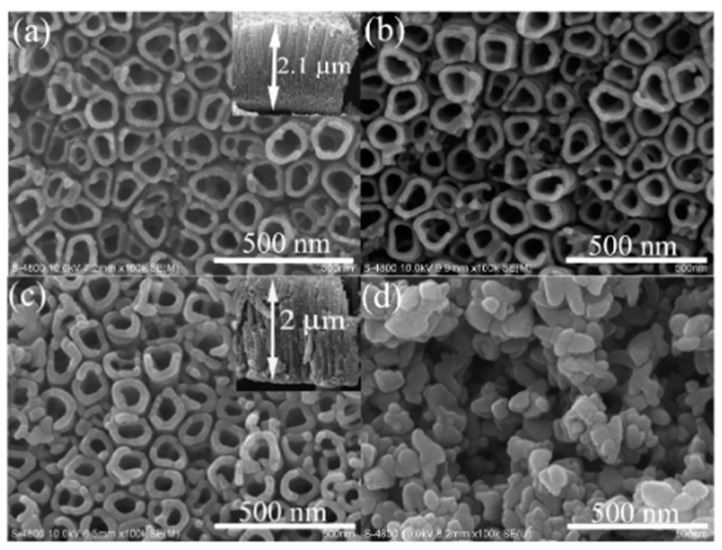
SEM images of the TiO_2_ nanotubes before and after the calcination procedure (**a**) untreated, (**b**) calcination, (**c**) vapor thermal, and (**d**) hydrothermal. Reproduced with permission from [87]. American Chemical Society, 2010.

Yu et al. [87] studied the effect of three different crystallization procedures on TiO_2_ nanotubes (Figure 7); samples calcined in a furnace at 450 °C retain the same morphology, pore diameter, and wall thickness as untreated TiO_2_ nanotubes (Figure 7a,b). The morphology of vapor thermal-treated TiO_2_ nanotubes at 180 °C changes; the wall thickness increases from 20 to 30 nm, while the inner diameter decreases from 70 to 50 nm. After hydrothermal treatment at 180 °C, the nanotubes are completely destroyed and transformed into aggregated particles with a size of approx. 100–150 nm.

## 5. Influence of the Various Crystal Structures of TiO_2_ Nanosurfaces on Bio-Performance

Titanium and its alloys, especially Ti-6Al-4V and NiTi (nitinol), are one of the most frequently used materials in cardiovascular, dental, and orthopedic applications, due to their excellent mechanical properties, corrosion resistance, and biocompatibility. However, the drawback is that these materials still lack the desired interaction with cells (rapid/selective proliferation of cells), as well as antibacterial activity. Many strategies have been proposed to produce more biocompatible surfaces, especially in the case of cardiovascular devices, like stents, where the selective proliferation of endothelial cells to smooth muscle cells should be obtained. The development was mainly directed at surface modification, such as coating techniques: ion implantation, electrochemical anodization, ion exchange, sol–gel techniques, hydrothermal treatment, plasma spraying, and the incorporation of metal ions such as silver, copper, or zinc [88,89,90]. With the growing field of nanotechnologies, much of the recent research has been devoted to the development of nanoparticles or nanostructures on the surface, as it was shown that different cell types react differently to specific nanostructures, as already mentioned herein. Thus, the nanostructuring of the surface could provide for cell selectivity if appropriately conditioned. Nevertheless, it is important to note that not only the nanostructure, but also other surface features like surface wettability, crystallinity, and chemistry, play an important role. It was already shown that prominent differences in osteoblast proliferation after 24 and 48 h of incubation were observed on TiO_2_ nanotubular surfaces, depending on their crystalline structure. The NTs, after heat treatment, transformed to an anatase structure, which seem to promote osteoblast proliferation compared to the amorphous freshly prepared nanotubes [72]. Another study by Lv et al. [91] reports that anatase TiO_2_ thin films led to better osteogenic activity in comparison with the rutile films with a similar film thickness, surface topography, and hydrophilicity. It was presumed that the enhanced osteogenic activity could be ascribed to the presence of more Ti-OH groups on the anatase film surface, which caused a more active conformation of the adsorbed fibronectin.

### 5.1. Influence on Osteoblast Cell Activity

The implants made from Ti and its alloys are widely used for orthopedic and dental applications, as they possess outstanding mechanical properties and biocompatibility. However, the main issue concerning these types of implants is their initial bonding with osteoblast cells (bone forming cells), which is relatively slow and another important issue concerning orthopedic implants is the prevention of bacterial adhesion. Many approaches for surface modification by mechanical/chemical treatment and the coating of surfaces with bioactive materials are employed, among the most common commercially available are plasma-sprayed biomaterial surfaces, coated surfaces with hydroxyapatite [92], and the production of microporous titanium surfaces, which provide more optimal support for osteoblast cell proliferation [93]. However, the desired biological response is not optimal, as high failure rates of implants in older patients or patients with compromised health are still observed. By the recent advances in three-dimensional printing (3DP) technologies, together with advances in computer-aided design (CAD) and computer-aided machining (CAM) processes, a new window for the fabrication of personalized medical devices was opened [94,95,96]. This further dictated the need for rapid and simple surface finishing procedures for custom-made implants, which will support more efficient osseointegration.

Although it is already well known that the chemical composition and topography of implant surfaces play a significant role in affecting the rate and extent of osseointegration [97], the crystal structure of TiO_2_ nanotube layers also seems to have an immense impact on cell proliferation and mineralization [86]. For instance, it has been shown that the proliferation and mineralization of osteoblast cells increased on anatase or a mixture of anatase⁄rutile TiO_2_ nanotube layers compared to amorphous ones [98]. Similarly, Bai et al. [86] showed the highest osteoblast cell activity on the TiO_2_ nanotubes with a rutile crystal structure. The reason for the increased osteoblast cell activity on crystallized TiO_2_ nanosurfaces could also play a role in the improved adsorption of protein functional groups that bond with cell surface receptors, which, among others, depends on the electrostatic properties of the surface [86].

The amount of proteins adsorbed on TiO_2_ nanosurfaces depends on surface characteristics, such as surface energy, wettability, topography, roughness, and surface charge [8,9,99,100,101], as well as the TiO_2_ crystal phase [102]. Hong et al. [103] showed that the hydroxyl groups on the surface of amorphous and TiO_2_ nanodots with an anatase crystal structure highly influence the adsorption of bovine serum albumin (BSA); BSA adsorbs as a monolayer on amorphous TiO_2_ nanodots but as a multilayer on anatase TiO_2_ nanodots. According to the authors, the reason for this is that the hydroxyl groups on the amorphous TiO_2_ nanodots attract more -NH^3+^ groups on the BSA molecules, causing the conformation of surface-bound BSA molecules to differ from those adsorbed on the anatase TiO_2_ nanodots. Fibronectin (FN), which plays a significant role in promoting osteoblast adhesion, is subsequently adsorbed on anatase TiO_2_ nanodots, on which it retains a more active conformation for further osteoblast adhesion and mineralization. Furthermore, a recently published study performed by Li et al. [12] showed that a higher number of Ti-OH groups on an anatase film surface caused a more active conformation of the adsorbed fibronectin. The authors suggest that fibronectin might adopt a more favorable conformation on the surface of an anatase–rutile mixture with higher anatase content.

**Figure 8:**
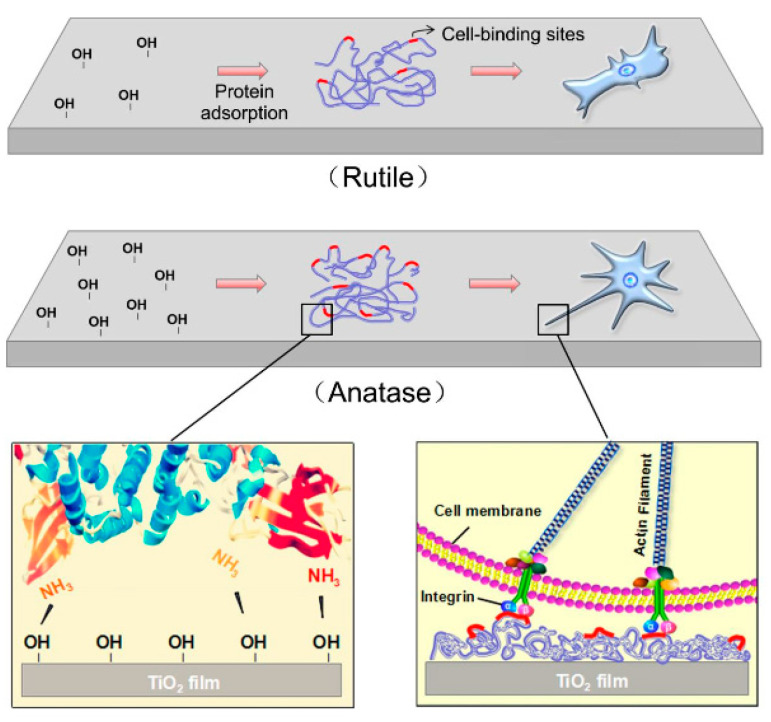
Schematic representation of the TiO_2_ crystal structure influence on osteoblast-binding site exposure of adsorbed proteins and osteoblast adhesion/proliferation. Reproduced with permission from [91]. Elsevier, 2017.

Lv et al. [91] also demonstrated that anatase TiO_2_ thin films promoted osteoblast adhesion, differentiation, and mineralization due to the presence of more hydroxyl groups on the anatase surface, which increased the cell-binding sites of FN exposed on its surface, causing a more active conformation of the adsorbed FN (Figure 8).

### 5.2. Influence on Hydroxyapatite Growth

The bioinert character of titanium makes this material widely accepted as the prerequisite for dental implants [104]. Surface modifications, creating micro-rough implant surfaces, even accelerate the desired osseointegration (bone–implant surface contact) of titanium [105]. Calcium phosphates, particularly hydroxyapatite (HA), are the principal minerals of the of hard tissues, such as bones and tooth enamel [106]. HA is therefore an indispensable element required for bone regeneration [107]. Various HA deposition techniques of metallic substrates have been employed on TiO_2_ surfaces, for instance, micro-arc oxidation and electrophoresis [108,109], plasma spraying [110], magnetron sputtering [111], sol–gel [112], and hydrothermal techniques [113].

Annealed TiO_2_ nanotube layers tend to induce HA growth, which is crucial for the successful bone bonding ability of the body implants [34,114]. However, there are disputed results on which surface features promote HA growth. Lv et al. [91] reported that a TiO_2_ film with an anatase crystal structure improves HA formation on metallic implants due to lattice matching and the superposition of hydrogen-bonding groups in anatase crystals compared to rutile crystals. Additionally, Jouanny et al. [115] showed that TiO_2_ thin films with anatase crystal structures are better candidates, as a TiO_2_ intermediate layer between hydroxylapatite and a Ti alloy, due to a reduced elastic modulus (120 GPa), which is close to the elastic moduli of hydroxyapatite bioceramic. On the contrary, Bai et al. suggest that a mixture of rutile and anatase in TiO_2_ nanotubes is even more efficient for promoting hydroxyapatite formation than a pure anatase phase [86].

### 5.3. Influence on Platelet Adhesion

**Figure 9:**
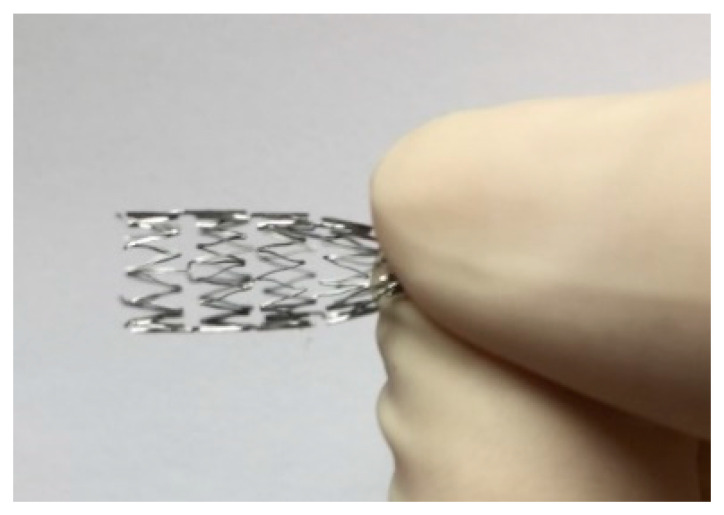
Bare metal vascular stent made from NiTi alloy (kindly donated by Rontis AG).

Cardiovascular diseases still present a serious health care problem and cardiovascular devices, like stents (Figure 9), are commonly employed in order to restore the blood flow through the diseased blood vessels. Although cardiovascular stents have saved countless lives, their surface properties still lack the desired biological response. The ideal medical device used as a vascular stent should promote the proliferation of endothelial cells (ECs), while at the same time reduce the proliferation of smooth muscle cells, which may cause a narrowing of the blood vessels (restenosis) and platelet adhesion (thrombosis). By the development of nanotechnologies, many different techniques have been employed to alter surface nanostructures in order to mimic the natural biological surface (biomimetic surfaces). It was shown that proteins and cells can detect objects much smaller than themselves and that different cell types react differently to the same nanostructure [20,93,116,117,118]. Thus, for specific applications, surfaces can be designed to elicit the desired biological response.

**Figure 10:**
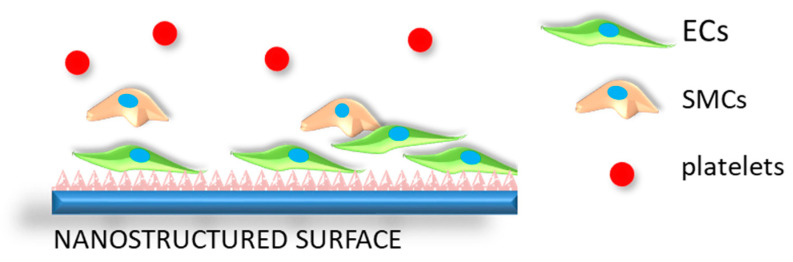
Schematic representation of the desired biological response of a nanostructured vascular stent surface in contact with the surrounding biological environment. The surface promotes the proliferation of endothelial cells (ECs), suppresses the adhesion of smooth muscle cells (SMCs) and prevents the adhesion, aggregation, and activation of platelets.

In the case of cardiovascular stents, the ideal nanostructured surface should promote the adhesion of endothelial cells and reduce the adhesion and activation of platelets. Additionally, the adhesion and proliferation of smooth muscle cells should also be suppressed, as shown schematically in Figure 10. Concerning topography, it should be noted that cells have a wide range of responses, which depend on many factors, including cell type, feature size, specific surface area, geometry, and the physicochemical properties of the materials. In a study by Smith et al., TiO_2_ nanotubes between 70 and 90 nm in diameter were shown to increase the adsorption of blood plasma proteins, the adhesion of platelets and their activation, and whole blood clotting kinetics [119]. Another study by Choudhary et al. reports that the adhesion of ECs and SMCs was improved for both cell types on the nanostructured surface compared to the conventional titanium surfaces. Furthermore, the EC growth was higher compared to that of SMCs [120]. According to Lu et al. [121], the nanometer topography offers a higher affinity toward adhesion, growth, and the alignment of ECs compared to micrometer-scale titanium patterns. Surface wettability, together with morphology, was studied Yang et al., where it was found that that superhydrophilic, as well as superhydrophobic, TiO_2_ nanotubes reduce platelet adhesion and activation compared to smooth titanium surfaces [122]. Another intriguing way to form nanostructured titanium surfaces is by hydrothermal treatment (HT), which allows for the synthesis of a stable, nanostructured coating made of anatase TiO_2_ crystals [14]. It was discovered that a metallic surface by hydrothermal treatment improves endothelialization and, at the same time, reduces smooth muscle cell proliferation [123]. Similarly, the hydrothermal treatment of titanium wires was employed to produce a nanotopography which, under static and dynamic conditions, showed negligible hemolysis, as well as inhibited the activation and aggregation of platelets. Moreover, the endothelium formed on nanostructured surfaces had an enhanced expression of antithrombogenic genes, providing for a longer coagulation cascade, probably due to a thicker oxide layer, in addition to topography [124,125].

**Figure 11:**
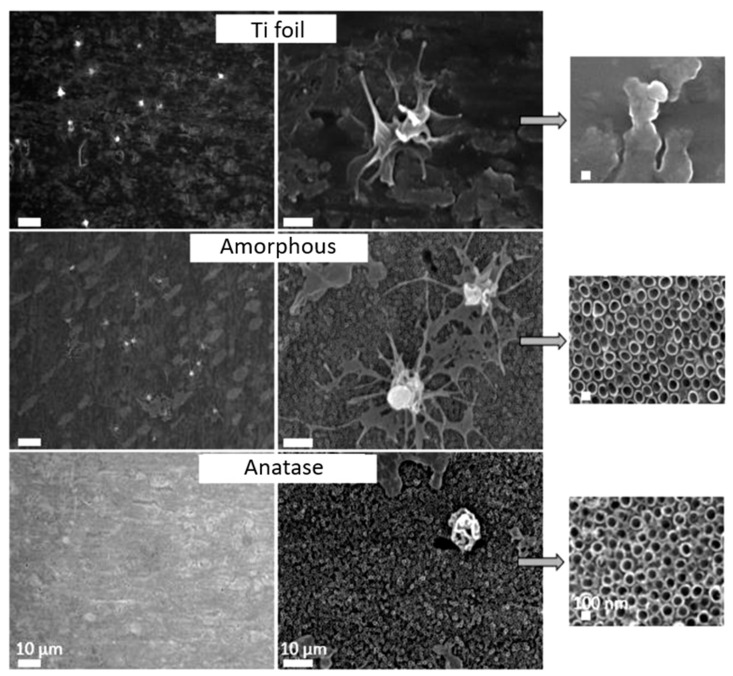
SEM images of Ti foil, amorphous NTs, and anatase NTs. Reproduced with permission from [73]. Materiali in tehnologije, 2019.

The recent results of our group indicate that not only topography, but also the crystal structure, of TiO_2_ nanosurfaces influence platelet adhesion and activation. The anatase TiO_2_ nanotubes (heat treated) were shown to reduce platelet adhesion and activation compared to freshly anodized amorphous TiO_2_ NTs and Ti foil [73]. In Figure 11. the interaction of whole blood with plain Ti foil, amorphous TiO_2_ NTs, and anatase TiO_2_ NTs with a 100 nm diameter is presented. It can be observed that on plain Ti foil, many platelets adhere and they are mainly in an activated form (spread and dendritic), a similar number of adhered platelets is observed on anatase NTs, and they have similar morphology. Interestingly, not many platelets can be detected on anatase NTs, and moreover, the platelets that can be detected are mainly in a round, and not activated, form. These types of materials could further provide the selective adhesion of endothelial and smooth muscle cells, as it was already shown in our previous study that nanotube surfaces after treatment with gaseous oxygen plasma enhance the proliferation of ECs and suppress the proliferation of SMCs [20,126].

## 6. Conclusions

Crystalline TiO_2_ nanosurfaces hold great promise in biomedical applications, since anatase and rutile phases often exhibit better bio-performance than the amorphous version of TiO_2_. Within the present review, the effect of various crystalline phases on selected biomedical applications is presented. In addition, this article discusses the most common crystallization techniques applied to TiO_2_ nanosurfaces. It presents intriguing methods for a rapid and feasible transition from amorphous to crystalline TiO_2_ phases. It is of significant importance that the crystallization process does not alter the surface morphology of TiO_2_ nanosurfaces, since it can dictate some constraints for the applicability of this material. For instance, it has been shown that various diameters of TiO_2_ nanotubes influence interactions with biological materials (proteins, cells) differently. However, most crystallization mechanisms affect the morphology of nanofeatures, especially in liquid/vapor media or long exposures to elevated temperatures. The oxygen plasma crystallization technique offers an elegant solution for the crystallization of TiO_2_ nanotubes, since it does not alter surface morphology. By changing plasma conditions, it is possible to crystallize amorphous TiO_2_ nanotubes into anatase, rutile, or a mixture of anatase and rutile crystal structures in a few seconds. Moreover, the beneficial effects on the proliferation of osteoblast cells and platelet adhesion are presented as a result of the surface-induced crystallization of nanostructured TiO_2_ medical devices.

## Figures and Tables

**Table 1:** Review of the low temperature plasma-induced crystallization of Ti-based nanomaterials.

Reference	Material	Initial Synthesis	Plasma Conditions
Ohsaki et al. (2009) [65]	TiO_2_ thin films	Sputtering/sol–gel	13.56 MHz RF, duration: approx. 2 min (a) oxygen plasma, 330 Pa, (b) Ar plasma, 330–2000 Pa
An et al. (2014) [66]	BaTiO_3_ thin films	Atomic layer deposition	Oxygen plasma (250 W, 15 mTorr) at 250 °C
Benčina et al. (2019) [33]	TiO_2_ nanotubes with 15 nm and 100 nm in diameter, Ti foil	Electrochemical anodization	13.56 MHz, 50 Pa, oxygen plasma, 400–800 W, duration 1–10 s
Xu et al. (2019) [63]	TiO_2_ thin films	Atmospheric pressure dielectric barrier discharges (AP-DBD) chemical vapor deposition	13.56 MHz, Ar plasma, 40–80 W, duration 30 min
Trejo Tzab et al. (2017) [64]	TiO_2_ powder	Sol–gel	Nitrogen plasma, power to max. 250 W, 60–120 min treatment, 30 Pa

**Table 2:** Comparison of different surface morphologies on plasma-induced crystallization in radio-frequency (RF) oxygen plasma at 800 W plasma power output and different treatment times (1 s and 10 s).

	Plasma Treatment		
Surface	Power (W)	Treatment Time (s)	Phase
Ti foil	-	-	amorphous
Ti foil	800	1	anatase/rutile
Ti foil	800	10	anatase/rutile
TiO_2_ NT 15 nm	800	1	anatase
TiO_2_ NT 15 nm	800	10	rutile
TiO_2_ NT 100 nm	800	1	anatase
TiO_2_ NT 100 nm	800	10	anatase/rutile

**Table 3:** Water contact angle (WCA) of Ti foil, amorphous, annealed, and plasma-treated TiO_2_ nanotubes (NT) with a 100 nm diameter. Plasma treatment P1 corresponds to a plasma output power of 200 W and a treatment time of 60 s, while P2 corresponds to a plasma output power of 400 W and a treatment time of 10 s.

Sample	Treatment	Crystallinity	Wettability—Water Contact Angle (°)					
			t = 0	Week 2	Week 4	Week 6	Week 8	Week 10
Ti foil	None	amorphous	97	98	94	99	97	98
**NT**	Electrochemical anodization	amorphous	4	11	6	26	58	73
**NT + heat**	Annealing (450 °C, 2 h)	anatase	2	10	8	19	18	32
**NT + P1**	Plasma treated (200 W, 60 s)	amorphous	5	8	16	18	51	48
**NT + P2**	Plasma treated (400 W, 10 s)	anatase/rutile	2	5	4	7	21	28

**Table 4:** XPS results of the atomic concentration of elements present on the surface of pristine Ti foil, nanotubes (NT diameters of 15, 50 and 100 nm) and nanotubes after plasma treatment at 200 W, 60 s (P), and nanotubes after heat treatment (annealing at 450 °C, 2 h).

Sample	C (at.%)	O (at.%)	Ti (at.%)	N (at.%)	F (at.%)
Ti foil	38.3	41.2	18	2.5	0
Ti foil + P	29.6	53.4	17.0	0.0	0
NT 15 nm	39.2	41.3	15.1	1.3	3.1
NT 50 nm	36.2	42.7	16.1	0.8	4.2
NT 100 nm	36.6	39.9	16.1	1	6.4
NT 100 nm + P	20.2	55.6	22.7	0	1.5
TiO_2_ NT 100 nm +heat	29.5	49.8	20.7	0	0
